# Transporting an Evidence-based Youth Development Program to a New Country: A Narrative Description and Analysis of Pre-implementation Adaptation

**DOI:** 10.1007/s10935-023-00742-2

**Published:** 2023-09-28

**Authors:** Finlay Green, Nick Axford, Ntale Eastmond, Vashti Berry, Julia Mannes, Kate Allen, Lynne Callaghan, Tim Hobbs

**Affiliations:** 1 Dartington Service Design Lab, Buckfastleigh, UK; 2 University of Plymouth, Plymouth, UK; 3 Mental Health Foundation, London, UK; 4 University of Exeter, Exeter, UK

**Keywords:** Adaptation, Implementation, Mentoring, Prevention, School, Violence

## Abstract

There is a pressing need to prevent and address youth crime and violence owing to its prevalence, harms and cost to society. Interventions with proven effectiveness in doing this exist. Adopting and adapting them in new contexts is potentially cost-effective. However, more research is needed into how to make adaptations that enhance intervention implementation, effectiveness and maintenance in new settings. This article reports the *pre-implementation* adaptation work involved in transporting Becoming a Man (BAM) from the US to the UK. BAM is a selective school-based youth development program for 12–18 year-old boys that aims to improve school engagement and reduce interactions with the criminal justice system. We describe the nature of and rationale for adaptations and identify learning for future adaptation efforts. An adaptation team comprising the intervention developers, new providers and the evaluators met weekly for 10 weeks, applying a structured, pragmatic and evidence-informed approach to adapt the BAM curriculum and implementation process. Changes were informed by documentary analysis, group-based discussions and site visits. The group agreed 27 changes to the content of 17/30 lessons, at both *surface* (e.g., cultural references) and *deep* (key mechanisms or concepts) levels. Of 28 contextual factors considered, 15 discrepancies between the US and UK were identified and resolved (e.g., differences in staffing arrangements). Strengths of the process were the blend of expertise on the adaptation team in the program and local context, and constant reference to and ongoing refinement of the program theory of change. Limitations included the lack of involvement of school staff or students. Further research is needed into potential conflicts between stakeholder perspectives during adaptation and whose views to prioritise and when.

**Supplementary Information:**

The online version contains supplementary material available at 10.1007/s10935-023-00742-2.

## Introduction

There is a pressing need to prevent youth crime and violence owing to its prevalence, harms and cost to society (Kieselbach & Butchart, 2015). Much is known about the effectiveness of school-, family- and community-based interventions designed to do this (e.g., Fagan and Catalano, 2013; Farrington et al., 2017; Matjasko et al., 2012; Russell et al., 2021). Those with the strongest evidence often originate in US, yet several have struggled to produce positive effects in Europe (e.g., Baldus et al., 2016; Fonagy et al., 2018; Humayun et al., 2017; Segrott et al., 2022; Skärstrand et al., 2013; Sundell et al., 2008). Reasons given for this include poor implementation, different context, lack of developer involvement and, of most relevance here, problems with program adaptation.

Adapting existing interventions for new contexts is potentially cost-effective because it saves investing in developing and evaluating new interventions (Movsisyan et al., 2019). Adaptation refers to a process of thoughtful and deliberate alteration of the design or delivery of an intervention to improve its fit or effectiveness in each context (Stirman et al., 2019). Whereas strict fidelity to intervention blueprints was once deemed essential to replication effectiveness, it is now recognized that reality is more complex (Chambers & Norton, 2016) and that staying true to function may be more important that adherence to form (Movsisyan et al., 2021). Making adaptations can improve program engagement, acceptability and outcomes (Stirman et al., 2019) but it can also go wrong, especially if changes remove or dilute active ingredients and thereby nullify the intervention theory of change (Evans et al., 2019; Movsisyan et al., 2019). Equally, a lack of adaptation can be unhelpful if incoming interventions inadequately fit the local service systems and culture (Moore et al., 2021). Moreover, lack of replication effect may be due to other reasons besides too much or too little adaptation, notably inflated evidence of effectiveness in the original study (Movsisyan et al., 2019).

It is generally accepted, then, that adopting an intervention in a new context requires making some changes to the intervention and context to achieve optimal ‘fit’ (Evans et al., 2019; Movsisyan et al., 2021). Done well, this can contribute to improved implementation, effectiveness and maintenance (Escoffery et al., 2018). A pragmatic, science-informed and stepwise but iterative adaptation process can help avoid ad hoc changes or program drift (Card et al., 2011; Evans et al., 2019; Moore et al., 2021; Movsisyan et al., 2019). Although several approaches to doing this exist, there is much agreement about fundamental principles (e.g., involving diverse stakeholders, agreeing a way of working, protecting the theory of change and core components) and steps (e.g., understanding the intervention and new community, consulting stakeholders, agreeing and making changes) (Escoffery et al., 2018; Moore et al., 2021; Movsisyan et al., 2019). However, there are few published case examples of the process in prevention and early intervention to improve youth psychosocial outcomes because adaptation is often done by practitioners dynamically during implementation rather than a priori.

One element of preventing youth crime and violence in the UK is to adopt and adapt evidence-based programs with this focus originating in other contexts. This article describes the pre-implementation adaptation process used with one such program that originated in the US and is now being implemented for the first time in the UK. Becoming a Man (BAM) is a selective school-based youth development program targeting 12–18 year-old boys. It is a program of Youth Guidance (YG), a Chicago (US)-based non-profit organization that provides school-based social-emotional and mental health programs across six cities in the US. It aims to improve school engagement and reduce interactions with the criminal justice system, doing so by helping boys to internalize six core values: integrity, self-determination, positive anger expression, accountability, respect for womanhood and visionary goal setting. The program comprises four core activities: BAM Circles (group sessions in school settings with 8 to 12 participants), special activities (group activities outside of school property/time), brief encounters (informal check-ins), and 1:1 support. The program is delivered by prosocial male counsellors who have QCF-6 level qualifications and receive 300 h of BAM training. BAM circles constitute the central element and involve 50 one-hour sessions over two years (~ 25 per year) made up of check-ins and check-outs to open and close sessions, role plays, group missions, video education, lectures, stories, and homework. Connections between BAM activities and desired outcomes are captured in the BAM theory of change, which was developed and adapted during the work described in this article (see below).

Two randomized controlled trials in Chicago, US, have shown positive impacts on numbers of arrests (for violent/all crime) and school performance for students with a mean age of ~ 15 years living in racially segregated and deprived communities (Heller et al., 2013, 2017). BAM has the highest rating on the Early Intervention Foundation Guidebook, the main UK registry for evidence-based programs, reflecting the quality of these studies and robustness of effectiveness results.^1^ In 2020, the Mental Health Foundation (MHF) introduced the program to the UK in three secondary schools in a south London borough. BAM was selected, based on evidence for its effectiveness, to help address a recent local increase in serious youth violence. It is being implemented with young people in school years 8 to 10 (ages 12–15 years). Funding for the project comes from the Youth Endowment Fund (YEF), a government-funded What Works Centre charged with preventing children and young people from becoming involved in violence by finding out what works and supporting efforts to put this knowledge into practice.^2^ A YEF-funded feasibility study and pilot outcomes evaluation undertaken by this research team (the authors of this article) aims to explore (i) its potential to improve outcomes in the UK and (ii) issues pertinent to further intervention delivery/development and a next-stage evaluation.

The adaptation process aims to enhance cultural relevance and build local ownership (Movsisyan et al., 2019). The pre-implementation adaptation work, which formed part of the feasibility study (Green et al., 2023), was informed by concepts and processes from existing guidance and frameworks (Card et al., 2011; Escoffery et al., 2018; Evans et al., 2019; Movsisyan et al., 2019; Stirman et al., 2019). The objectives of this article are to (i) describe narratively the pre-implementation adaptation process and the nature of and rationale for the adaptations made, and (ii) reflect critically on the strengths and limitations of the process and identify learning for future adaptation efforts.

## Adaptation Process

Before making any program adaptations, the research team articulated the BAM theory of change to help guide the adaptation process and evaluation (see Green et al., 2023). This combined evidence and theory from academic literature selected by YG with local (US, UK) stakeholder expertise to articulate high-level predictions about how BAM is supposed to work, for whom, under what circumstances and why. This included: published qualitative (Lansing et al., 2016) and quantitative (Heller et al., 2013, 2017) research about BAM; theoretical frameworks for group therapy (Yalom & Leszcz, 2005), psychotherapy (Jung, 1969), youth development (Nagaoka et al., 2015), and behavior change (Michie et al., 2014), which together form the foundations of BAM’s approach to ‘action’ and ‘reflection’; systematic reviews of community-based positive youth development interventions, which are similar in their theoretical foundations to BAM, as well as reviews of process evaluations and theories of change for these interventions (Bonell et al., 2016); reviews of program documents, including the BAM circle curriculum; the observation of BAM circles in Chicago; and workshops and interviews with staff from YG and MHF.

Articulating BAM’s underlying mechanisms was an important part of this process. However, evaluations based on Theory of Change can sometimes overlook or under-examine the core functions of interventions (Breuer et al., 2015). This is a risk that Realist Evaluation is well-placed to mitigate, given that it offers principles and practices that help evaluators to interrogate causal relationships (Blamey & Mackenzie, 2007; Rolfe, 2019). The research team therefore adopted Realist Evaluation’s definition of a mechanism during development of the theory of change: an explanation for how a particular set of program resources leads to a response in stakeholders’ reasoning (Dalkin et al., 2015).

Pre-implementation adaptations to BAM then proceeded using a twin-track process focused on curriculum and implementation respectively. First, adaptations were made to the BAM curriculum. This is contained in a 30-lesson manual covering the content that should be delivered in the BAM circle (the main program component) over two years. An adaptation team comprising representatives from YG (n = 5; operational, replication and evaluation expertise), MHF (n = 6; program management, delivery and evaluation roles) and the research team (n = 1; program adaptation and evaluation expertise) was set up to work through the curriculum lesson-by-lesson and make necessary adaptations. There were 10 weekly online adaptation sessions between July and October 2020, each lasting two hours and covering approximately three lessons.

The delivery partners (MHF, YG) owned and led the curriculum adaptation process, with adaptation decisions arrived at by consensus. The research team documented the process and results to help with evaluating adaptations. This included collecting information that would allow us to describe and categorize adaptations and identify factors that might influence their feasibility. To support this, we drew on existing guidance (see above) to develop a framework comprising seven areas: (i) target (what is adapted?); (ii) nature (how is content adapted?); (iii) rationale (why is it adapted?); (iv) degree (how much is it adapted?); (v) agents (who did the adapting?); (vi) implications (what else needs adapting?); and (vii) effect on the theory of change (is the theory of change adapted by default?) (Appendix A).

We created a matrix based on this framework to record each change made to the curriculum. Some elements of the framework were known in advance and therefore did not need recording, notably the agents involved in making the adaptations, and it was agreed that others, such as target and nature of changes, could be recorded *post hoc* based on key information about the changes made. Thus, the matrix comprised these categories: (i) lesson (1 to 30); (ii) adaptation (a description of the change agreed); (iii) rationale; (iv) whether the adaptation was surface-level or deep,^3^ including its effect on the BAM theory of change; (v) whether other adaptations were needed because of the adaptation in question (Yes/No, explanation); and (vi) the date the adaptation was agreed. The MHF Research Manager for BAM attended all curriculum adaptation sessions and documented changes in the matrix.

The second set of adaptations concerned implementation. The contexts of London and Chicago differ in many ways, from the challenges youth face and the nature of the school environment to the wider systems and communities within which youth and schools are embedded. Within BAM, there are a series of ‘implementation teams’ designed to ensure that these contextual factors support rather than impede the implementation and impact of BAM. To increase the likelihood of a good intervention-context fit, it was necessary to consider differences in these wider contextual factors and their implications for the responsibilities and activities of BAM’s implementation teams. The context areas we considered were drawn from the BAM theory of change and relevant literature. They cover features identified by Craig et al. (2018): epidemiological; social and economic; cultural; geographic / environmental; service / organizational; ethical; policy; legal; financial; political; historical; and external shocks / cataclysmic events. They also cover the ‘inner setting’ and ‘outer setting’ domains in the Consolidated Framework for Implementation Research (Damschroder et al., 2009, 2022).

Bearing these in mind, the adaptation process for implementation involved the following: (i) describing the situation in the original (US) implementation setting(s); (ii) identifying and explaining the factors hypothesized to influence outcome variation in the original setting; (iii) providing evidence of *contribution or causation* to support this hypothesis in the original setting(s); (iv) describing the situation in the new setting(s); (v) identifying discrepancies between settings and explaining why they need resolving; (vi) describing and justifying the adaptation in response; and (vii) ensuring that adaptations align with the BAM theory of change.

As with curriculum adaptation, we created a matrix to document changes. This was populated using several data collection methods: analysis of program documentation and studies; a visit by a member of the research team (FG) to Chicago to observe BAM delivery in schools and meet stakeholders; and interviews and group-based discussions involving MHF and YG staff (developers, trainers, local implementers, other relevant stakeholders). The process was iterative to allow opportunity for refinement as new information emerged.

## Curriculum Adaptations

A total of 27 changes were made to the content of 17 out of 30 lessons. Surface adaptations are described in Table 1. Deep adaptations fell into three categories.

**Table 1:** Surface adaptations to the BAM curriculum

Nature of adaptation	Example(s)
Superficial changes to language, where the reference or meaning of the word(s) remains intact.	In a session on accountability, the counsellor shares a story with students about the actions of a travelling salesman who gets lost. This prompts a discussion about being accountable for one’s actions. In the story, distance is denoted in terms of ‘city blocks’, which in the UK version became ‘streets’.
Changes to cultural references, where both language and reference change but the purpose and function of the reference remain the same.	In a session about self-determination, having ‘basketball tryouts’ is cited as a reason young people might give for attending school. In the UK version, ‘basektball’ was replaced with ‘football’. In a session about integrity, there is a role play in which one student borrow money from another student but never returns it. The amount borrowed ($10) was changed to £10. Films that may resonate more with a US audience were replaced with those deemed better suited to London youth. For example, in a session on self-determination students are shown clips from the film *Miracle*, focusing on characters in the US ice-hockey team who push themselves to achieve their goals. This was replaced with clips from the film *Pursuit of Happyness*, which is about a man who experiences homelessness before becoming a successful stockbroker.
Amending the timing and structure of sessions on ‘academic integrity’^1^ to account for differences in the assessment processes between the two countries	Aligning sessions with the release of grades throughout the school year.
Adapting to UK COVID-19 restrictions	Ensuring students use hand sanitizer prior to group sessions.
Replacing the language used during ‘check-ins’ with terms more commonly used by London youth (every session should include at least one check-in, where the group takes turns to describe how they are feeling and why).	Certain rituals occur during check-ins, the most important being the way the group responds to someone checking in by saying “Asé”, which means “I’m with you” or “I hear you” in Yoruban language. In London, this was replaced with “safe”, “say less” or “calm”, colloquialisms common in London and used, among other things, as greetings.

^1^ During academic integrity sessions, participants take turns to update the group on their most recent grades. The group then affirms those students who have passed all their classes, and challenges those who have failed all or some of their classes.

### Tribal Societies

BAM is designed to be a two-year rite of passage from boyhood to manhood. The concept is a spiritual one, in the sense of encouraging young people to connect to something bigger than themselves. In BAM, this ‘something’ is the universal experience of transitioning into manhood. BAM frames rites of passage as coming from and being grounded in ‘tribal’ societies, and uses various archetypes throughout the curriculum to highlight their timeless nature. Archetypes are models of people, behaviors or personalities which have universal meanings across cultures (Jung, 1969). They naturally attract or repel young people and provide shortcuts, helping youth to learn and internalize a concrete manifestation of what each value is and is not, rather than offering an abstract assortment of defining features. Here, the connection to something bigger makes the journey to manhood meaningful and important, which motivates young people to stay the course and practise the core program values.

Upon review, MHF and YG saw that the phrase ‘tribal societies’ is problematic, and both organizations sought to change the language. In particular, MHF felt that the term ‘tribal’ had problematic colonial and imperialist associations in the UK context. Specifically, the phrase was emblematic of the Eurocentric tendency to inappropriately group together alternative cultures and lifestyles as ‘tribal’ and overlook the differences between them. Consequently, references to ‘tribal’ were replaced with ‘communal’. Both YG and MHF felt this would allow the concept of rites of passage to retain its historical significance, while allowing participants to bring their own histories to the idea.

### ‘Savage’ and ‘Warrior’ Energy

The ‘savage’ and the ‘warrior’ are examples of additional archetypes used in BAM, here as an important mechanism of change. During sessions on Positive Anger Expression (one of six core values), ‘savage’ is used to conjure up images of destructive, uncontrolled anger that creates guilt and shame, while ‘warrior’ is associated with constructive, controlled anger that brings dignity and integrity.

Upon discussion, MHF felt these concepts were problematic. First, both have violent connotations, which could unintentionally suggest that anger equates to violence. Second, ‘savage’ has been used historically as a derogatory term for indigenous peoples. Third, ‘savage’ is already used as a colloquialism among youth in London, often as a compliment to imply strength. As a result, MHF and YG agreed to use the terms ‘constructive’ and ‘destructive’ as archetypes.

### The ‘Liberator’ and the ‘Oppressor’

The two most important archetypes introduced during the ‘Respect for Womanhood’ core value are ‘self-liberator’ and ‘oppressor’. The former is associated with men who share their power with women and the latter with men who use that power to weaken or subjugate women. MHF felt that the term ‘liberator’ did not challenge the fundamental issue that men hold power in the first place and risked reinforcing the idea that women are weak and only gain power when men grant it to them. As a result of this discussion, YG corrected an error in the BAM curriculum, ensuring use of the term ‘self-liberator’ – to reflect that it is not an action being ‘done to’ women but rather a change that must take place internally for men (i.e., not to ‘liberate’ women, but to liberate themselves from negative perceptions or stereotypes of women). It was also agreed to further emphasize the meaning of that term during the curriculum.

## Implementation Adaptations

Prior to implementation, 28 contextual factors were identified, with potential discrepancies between BAM’s US^4^ and London contexts documented for each (Table 2). For five of these, no discrepancies were identified. For 15, discrepancies were identified and resolved. For seven, it was unknown whether there would be discrepancies because the project was in its infancy.^5^ For the final factor, discrepancies were identified but not addressed pre-delivery (since partially addressed).

**Table 2:** Categorization of adaptations to implementation

	Broad category^1^	Specific category^2^
**No discrepancy** [n=5]		
Counsellor ethnicity	Delivery	Demographic
Counsellor age	Delivery	Demographic
Counsellor gender	Delivery	Demographic
Match counsellors to school	Implementation teams	Selection of counsellors (pre-implementation)
Marketing to avoid negative labelling	Implementation teams	School implementation team
**Discrepancies identified and resolved** [n=15]		
Teacher strikes	Delivery	External shocks
Winter weather	Delivery	Geographical / environmental
Background checks on counsellors	Delivery	Legal
Spirituality	Delivery	Cultural
Routine data	Implementation teams	EQI team
Viability of brief encounters (COVID-19)	Delivery	Service / organizational
Remote delivery (COVID-19)	Implementation teams	BAM Training Academy
School staff energy (COVID-19)	Implementation teams	School implementation team
Counsellor recruitment (COVID-19)	Implementation teams	Selection of counsellors (pre-implementation)
Counsellor training/coaching (format, length, frequency) (COVID-19)	Implementation teams	Core team
School-level agreement	Implementation team	School implementation team
Counsellor recruitment	Implementation teams	Selection of counsellors (pre-implementation)
External champions	Delivery	Political
Engagement / collaboration with other services	Delivery	Service / organizational
Parent engagement	Participation	Social / economic
**Unknown pre-implementation** [n=7]		
Stakeholder attitudes towards masculinity	Delivery	Cultural
Rivalry with other local youth work organizations	Delivery	Historical
Counsellor competencies	Delivery	Service / organizational
Counsellor turnover	Delivery	Service / organizational
Participants – demographic profile	Participation	Demographic
Participants – epidemiological profile	Participation	Epidemiological
Advisory Council	Implementation teams	Advisory Council
**Identified but not addressed** [n=1]		
Clinical supervision	Implementation teams	Core team

^1^ Delivery context; Participation context; Implementation teams

^2^ Type of (a) context and (b) implementation team

### No Discrepancies

There are two broad areas where no discrepancies were found. First, the counsellors’ profiles are largely the same in terms of ethnicity (African American/Black British), gender (male), age (no younger than the age at which most people graduate college) and the extent to which they were matched with schools; counsellors who work with more challenging youth in the US must have prior experience of working with these groups, a policy adopted in the UK for the counsellor working in a Pupil Referral Unit.^6^

Second, when recruiting young people, YG ensures that: (i) BAM is marketed as a social-emotional program to help all youth, not a behavioral program for those displaying behavior problems; and (ii) the cohort presents with a range of social-emotional strengths and challenges, including externalizing and internalizing concerns. This helps to prevent both negative labelling and any sense among students that BAM somehow ‘rewards’ anti-social behavior. These policies were adopted in the UK.

### Discrepancies Identified and Resolved

#### Problematic in the US, but Not in the UK

Five contextual factors identified as previously occurring implementation barriers for BAM in the US were considered less salient in the UK, requiring no action. First, while teacher strikes can be frequent in the US, they are less common in the UK (at least in the recent past). Second, severe winter weather, which can regularly interrupt delivery in the US, is less of a barrier to implementation in the UK. Third, implementation delays have occurred in the US due to difficulties with counsellors clearing background checks in particular school districts, but counsellors in London were registered and cleared to work with minimal difficulty. Fourth, stakeholder attitudes to the role of spirituality in BAM were deemed less problematic in the UK. In the US, local government partners in some sites had concerns that the spiritual connotations of ‘rites of passage’ might jeopardize the separation of religion and state in school. In the UK, by contrast, it is more common for state schools to be associated with religion, indeed one participating school is faith-based. Finally, the extent to which counsellors and implementation support staff participate in interpreting and acting on routine data is variable in the US, but less of a concern in the UK. This is partly because the Client Management System in London is more flexible than that used in the US, so MHF can tailor it to counsellors’ needs. Additionally, the MHF research manager has more time to support counsellors than would be normal for her US equivalent (because currently there are only three counsellors in the UK).^7^

#### COVID-19-related Adaptations

The COVID-19 global pandemic and associated lockdown restrictions (including school closures) coincided with the feasibility phase of the BAM evaluation and therefore needed to be addressed in the pre-implementation phase. The first COVID-19-related adaptation concerned ‘brief encounters’, incidental, informal and unscheduled contacts between youth and counsellors that happen spontaneously during the school day. These usually occur between classes in a communal space in the school building, although they can include a student dropping into the BAM room to talk with a counsellor. They allow BAM participants to practise core values in different contexts and settings, while also supporting the development of the participant-counsellor relationship. YG typically expected counsellors to reach 80% of their caseload per week with brief encounters. Due to COVID-19, however, it was difficult for counsellors to spend long periods of time in indoor communal spaces, so they were encouraged to engage young people in brief encounters in other safe spaces (e.g., the playground). As this was not mandated during COVID-19 and no ‘minimum level’ was required of counsellors in that period, fewer brief encounters were expected.

The second COVID-19-related adaptation concerned the mode of delivery. While London was delivering BAM in-person, US sites were delivering online. To facilitate this, the BAM Training Academy designed a curriculum to keep students engaged through online means during this challenging time (i.e., session plans delivered via video calls). While counsellors in London were able initially to deliver face-to-face in schools, the online curriculum was available for when students were unable to attend school for a prolonged period (3–4 months depending on the school and group).

The third adaptation triggered by COVID-19 concerns schools. It is important that school leadership prioritizes and champions BAM, otherwise teachers may be less likely to let students leave class for BAM sessions or to consider BAM’s needs when making decisions that impact on BAM (e.g., regarding which activities take priority for limited classroom space). The energy needed to adapt to COVID-19 prevented school staff from being able to prioritize BAM. MHF responded by supporting schools’ COVID-19 response. This included developing mental health resources for teachers and students and supporting the implementation of COVID-19 restrictions in school. It was anticipated that fostering positive relationships in this way would demonstrate that counsellors are core members of the school community and also mitigate the impact on BAM of schools’ focus on COVID-19.

Finally, COVID-19 restrictions meant that counsellor recruitment interviews, training and coaching were moved online. Training and coaching sessions were shortened (two hours rather than a half or whole day) and held more frequently (every week rather than once a month) to accommodate this change in format. Group observations that are a normal part of coaching activities did not occur.

#### Responding to BAM’s Lack of Profile in London

In the US, BAM was publicly championed by then President Obama and has a strong evidence base. This makes it an attractive prospect for school districts, communities and parents, helping to overcome indifference or resistance. No such platform exists in the UK. Moreover, various actors already influence the issue of youth violence in London, many of which will shape or be shaped by BAM in ways that will affect delivery. MHF felt that it was essential to capitalize on this network of actors or risk antagonizing them in ways that might impede implementation. Consequently, it decided to proactively develop relationships with key stakeholders. This had three elements.

The first concerns schools. School Implementation Teams represent a formal partnership between school leadership and BAM. Their role is to ensure that each school is an enabling context for implementation and to use data to inform program-related decisions. In the UK, the School-Level Agreement^8^ was amended to make the language more approachable and less direct (e.g., removing legalese). This was partially due to differences between countries in school governance, with schools in the UK having more delegated authority than their US counterparts to make decisions about their own provision. Additionally, schools were directly involved in recruiting BAM counsellors, something that YG had never done.

Second, MHF established formal partnerships with two respected community organizations in London who now support implementation. Black Thrive and Colourful Minds support MHF to develop positive relationships with participants’ local communities, including their families and other organizations and individuals in the local service network.^9^ The aim is to ensure that their respective efforts are complementary.

Third, MHF decided to hold open parents evenings early in the school year to share information about BAM. In the US, counsellor engagement of parents has tended to be more discretionary.

#### Staffing Discrepancies

In the US, supervisors provide day-to-day administrative, project management and co-ordination support. This includes taking an active role in multiple implementation teams, notably the school implementation team. In BAM’s replication sites (Boston, Los Angeles, Seattle), the supervisor’s role is usually supplemented by a senior staff member from the local delivery organization, who undertakes more external-facing and strategic responsibilities. For example, they are a member of the Leadership Team and often responsible for establishing and maintaining the Advisory Council (see below), two important implementation teams. In London, MHF recruited a project manager as the supervisor. The commitment and skill this individual demonstrated meant that he assumed more responsibility than would normally be expected of a supervisor, including being a central figure in the Leadership Team.

### Possible Discrepancies (Unknown Pre-implementation)

#### Perceptions Held by Other Organizations

Prior to implementing BAM in London, it was unclear whether there were discrepancies in the perceptions of BAM held by other stakeholders in the local community (e.g., whether they endorse or object to BAM’s focus on male identity, or view BAM as a threat). These perceptions can influence implementation by reinforcing or undermining young people’s sense of belonging to BAM (an important mechanism in the theory of change). Since then, it has been established that the wider community and other institutions broadly support BAM’s focus on boys and masculinity (certainly no less than would normally be expected in the US). Moreover, counsellors have not reported rivalry with other local youth work organizations when asked, despite historical underfunding of the sector locally.

#### Counsellor Competencies and Retention

When implementation was in its early stages, it was impossible to say whether discrepancies exist regarding the counsellors recruited in London and those delivering BAM in the US. For example, the baseline level of competency of London BAM counsellors had not been assessed, nor was it clear how counsellor turnover might affect implementation. Since then, no discrepancy has been identified in either case; London BAM counsellors’ levels of competency (in particular youth engagement skills) are in the normally expected range in the US, albeit at the upper end of the spectrum, and there has been no counsellor turnover.

#### Participants

Prior to completing selection, it was not possible to determine whether discrepancies existed between London and the US regarding the demographic and epidemiological profile of BAM youth because the data did not exist. We now know that the demographic profile (i.e., age, ethnicity) of UK BAM students is within the range of BAM students in the US. However, .owing to a lack of data at the time of writing, it remains unknown whether a discrepancy exists for offending and school engagement.

#### Advisory Council

A local Advisory Council is established in each region where BAM operates. This includes local community and regional/national industry leaders. Their role is to act as a two-way liaison with local communities, promote the financial sustainability of BAM, and identify programmatic opportunities for young people. As the Advisory Council was not established in London prior to delivery, it could not be compared with other BAM sites (this remans the case). One reason for this is that it did not seem a priority in the early stages of program implementation given the limited capacity of the adaptation team, challenges related to COVID-19 and the fact that the program was already fully funded for two years. Related to that are differences in funding sources for such interventions; the US has a stronger culture of individual giving and donations from large trusts, foundations and corporates, whereas in the UK it was felt that a more sustainable and realistic funding model would be via statutory bodies responsible for planning and commissioning local health care services. It is also the case in the US that the Advisory Council is typically established once the program is running.

### Discrepancies Identified but Not Addressed

In the US, Curriculum Specialists support counsellors to develop their competencies and deliver the curriculum with quality and fidelity. However, in the UK, clinicians must receive regular clinical supervision. In the planning stage, therefore, it was intended to add a clinical supervisor to the Core Team (the primary implementation team) in London, to supplement support provided by the Curriculum Specialist. This did not happen; a decision was made collectively that supervision from the program manager and the Curriculum Specialist together was sufficient.

## Critical Reflection and Learning

The aim of this article was to report the *pre-implementation* adaptation work associated with transporting an evidence-based program from the US to the UK. The first objective was to describe the adaptation process and the nature of and rationale for the adaptations made. Adapting the curriculum involved a 10-week group process led and owned by the purveyor and provider, with the research team recording changes, specifically what was changed, why, when, if and how it affected the theory of change and whether it required further changes. The group agreed 27 changes to the content of 17/30 lessons, at both *surface* (e.g., cultural references) and *deep* (key mechanisms or concepts) levels. Adaptations to facilitate effective implementation in a new context entailed analysis of BAM documentation and studies, a site visit to the US and interviews/discussion with the purveyor and provider. Changes were recorded, focusing on the contextual situation in the original (US) setting and its influence on outcomes, the situation in new setting, potential discrepancies in need of resolving, and the nature of and rationale for any change. Of the 28 contextual factors examined, discrepancies identified and resolved (n = 15) related to implementation barriers in the US that do not apply in the UK, COVID-19-related issues, the lack of profile for BAM in the UK and differences in staffing arrangements. For some discrepancies (n = 7) it was too early pre-implementation to say if they would be problematic, namely perceptions held by other organizations, counsellor competencies and retention, participant profile and the Advisory Council. Some contextual factors were deemed not to have discrepancies between settings (n = 5) and one could not be addressed pre delivery.

The second objective, which is the focus of this section, was to reflect critically on the strengths and limitations of the process and identify learning for future adaptation efforts. The context for this is that complex psychosocial interventions are difficult to design, implement and evaluate. Transporting them from one context to another adds another layer of complexity, with many such efforts resulting in null effects when trialled. Making too many or too few changes to the intervention, or failing to appreciate and address important contextual differences, are among the reasons given for such results (e.g., Movsisyan et al., 2021). Although often neglected, it is therefore important to document intervention adaptations and explore their impact on acceptability, implementation and outcomes (Chambers & Norton, 2016; Escoffery et al., 2018; Stirman et al., 2019). There is also a pressing need to document how adaptation guidance is used and reflect on its usefulness (Copeland et al., 2022). Cumulatively, such studies will further knowledge about how to adapt well and inform adaptation strategies (Chambers & Norton, 2016; Moore et al., 2021).

This article contributes to this endeavour by describing the process and results of the *pre-implementation* adaptation phase for an evidence-based intervention originating in Chicago and now implemented in London. As such, it captures the complexities and challenges of real-world intervention adaptation. Like the majority of studies describing or evaluating adaptation projects in health services and public health looked at by Movsisyan et al. (2021), the current study focused on a micro-level intervention, but in other ways it helps to address deficiencies in the evidence identified in that review. Unusually, it concerns a cross-continent adaptation (most such studies are within-country, especially in the US), describes the quality of evidence that informed program selection in the new setting and is specific about the rationale for different adaptations. Further, the program theory of change was central to the adaptation effort, as was consideration of multiple aspects of context in the new setting and how they or the program might need to be adapted to achieve an optimal fit. Also in contrast to studies in the Movsisyan et al. (2021) review, the adaptations made and described here were less about program content and more about implementation context.

We applied a pragmatic, evidence-informed approach, borrowing concepts and steps from established adaptation guidance which, as others have found, proved useful but too long and time-consuming to apply in full (Copeland et al., 2022). This likely explains why reported adaptation efforts in health services and public health tend not to use guidance religiously but do adopt a structured approach that is in line with guidance, underpinned by key principles and following a sequential process (Movsisyan et al., 2021). The method was efficient and effective in identifying issues requiring attention and making requisite adaptations to the curriculum and implementation process.

Two features of the adaptation process arguably contributed to its success (measured in those terms), starting with the blend of expertise on the adaptation team in the program and local context. The developer/purveyor (YG) listened respectfully to local concerns from the delivery organization (MHF) and its practitioners about possible mismatches between settings and helped to make context-sensitive adaptations that preserved core program elements. This was facilitated by similar organizational cultures across YG and MHF. The participatory approach to adaptation was not explicitly informed by theory, unlike some adaptation projects which have deliberately drawn on community-based participatory research principles (Movsisyan et al., 2021). Nevertheless, the work did adopt a partnership approach, with equitable involvement from all parties (developer, implementers, researchers) and a strong sense of shared decision-making and joint ownership. Other studies have reported the benefits of this for program implementation, including increased acceptability, responsiveness to local needs and likelihood of sustainability (ibid.). Collaboration is not without challenges, of course, and further research is needed into potential conflicts between stakeholder perspectives and whose views to prioritise and when (ibid.).

The other aspect of the process that supported effective adaptation was the development and ongoing refinement of the BAM theory of change. This allowed adaptation conversations to be well structured, acting as a constant reference point and informing both (i) the focus and substance of discussions and (ii) decisions about the nature of and rationale for changes to the curriculum or implementation. This helped to maintain consistency with intervention functions, alongside our use of Realist Evaluation’s clear and widely-used definition of program mechanisms. By contrast, none of the studies reviewed by Movsisyan et al. (2021) reported on program theories of change or modifications to them, or reflected on the importance of cultural or structural factors for intervention mechanisms and how they may interact with such mechanisms to affect implementation and outcomes in new settings. Our experience did not suggest the need for significant changes to existing adaptation guidance or frameworks in this respect, although we found it helpful to consider explicitly theories or evidence of how implementation strategies used in the original setting contributed to outcomes.

Inevitably the pre-implementation adaptation process also had limitations. First, the intervention was selected prior to our involvement, so we could not undertake common pre-selection analyses of fit with local needs. Second, owing to COVID-19 restrictions, MHF staff were unable to make an intended trip to the US to observe BAM in practice prior to implementation in the UK and were therefore reliant on written and third-party accounts of the program in action. Third, COVID-19 meant that some aspects of the implementation process had not been established before delivery commenced and so possible mismatches could not be identified upfront. Fourth, the focus on adapting *existing* implementation approaches meant that new or alternative strategies were perhaps not considered sufficiently. Finally, school staff and young people were not involved in pre-implementation adaptation. They were, however, involved in data collection later in the feasibility phase evaluation and their insights about the experience of delivering and receiving BAM respectively contributed to further adaptations (Green et al., 2023).

As delivery continues in London, the intervention and BAM’s implementation teams continue to change; initial adaptations are evolving and new ones emerging. We are testing intervention feasibility and potential impact on outcomes and continue to work with partners to record and evaluate adaptations, with feedback loops contributing to ongoing adaptation. The focus is primarily on ‘deep’ rather than ‘surface’ adaptations, with the aim of establishing (i) the alignment of these adaptations with their core function, and (ii) the acceptability, appropriateness and feasibility of their form. An interesting question is whether BAM has been adapted for London or for the UK, the answer to which will only become apparent if and when it is adopted elsewhere in the UK. Further research is needed to explore whether, as we suspect, deep adaptations to interventions are likely to apply at a country level and therefore not need further significant adjustment, whereas more localized differences in culture, service organization, and geography will require additional surface adaptations.

### Electronic supplementary material

Below is the link to the electronic supplementary material.

Supplementary Material 1

## Footnotes

1 A Level 4 rating requires evidence from at least two high-quality evaluations demonstrating positive impacts across populations and environments lasting a year or longer. https://guidebook.eif.org.uk/programme/becoming-a-man.

2 https://youthendowmentfund.org.uk.

3 The distinction between the two rests on whether an adaptation interferes with the core functioning of the programme.

4 BAM is now delivered in US sites besides Chicago, notably Boston, Los Angeles and Seattle.

5 Of these, subsequently, for five no discrepancy has been identified, at least beyond the normal range of variation in the US, for one some discrepancies have been identified and addressed, and for one the level of discrepancy remains unknown.

6 A PRU is a form of alternative educational provision for children who cannot attend mainstream school owing to behavior which would disrupt other students.

7 The task of supporting the team with data and related activities will become more challenging as additional schools are added in the UK.

8 A contract between the school and BAM which details the responsibilities of each.

9 Black Thrive aims to address the inequalities that negatively impact on the mental health and well-being of Black people in the London borough where BAM is being implemented: www.blackthrive.org. Colourful Minds aims to improve public understanding of mental health and mental illness through education in the Black and minority ethnic community: www.colourful-minds.org.uk.
