# Supplementary material for: Transporting an Evidence-based Youth Development Program to a New Country: A Narrative Description and Analysis of Pre-implementation Adaptation
Source: J Prev (2022). 2023 Sep 28;44(6):729–47. doi: 10.1007/s10935-023-00742-2 (PMC10638196; doi:10.1007/s10935-023-00742-2)
Supplement: Supplementary file 1 — Supplementary Material 1 [file 10935_2023_742_MOESM1_ESM.docx]

# APPENDIX A: Adaptation framework – key issues to consider

**1. Target (what is adapted?)**

Structure

- order
- strands

Content

- language (actual, literacy level, culture)
- research-based information e.g., statistics
- images / examples (up-to-date, culturally relevant)

Delivery

- agencies
- agents/personnel/staff
- format

Context

- location
- setting
- inner/outer

Recipients

Training/support

**2. Nature of adaptations (how is content adapted?)**

Adding

Removing

Lengthening

Shortening

Refining

Reordering

Repeating

Integrating

**3. Rationale (why is it adapted?)**

Accuracy

Fit to context / salience

Acceptability / local ownership and commitment

Engagement (enrolment, retention)

Satisfaction

Sustainability

Effectiveness

Cost

+ Based on what analysis/information (and by whom)

**4. Degree (how much is it adapted?)**

Surface / deep

Core components / discretionary components

[As far as possible aim should be to retain core components / retain deep structure]

**5. Agents (who did the adapting?)**

Local community leaders, partners and implementers

Representatives of the target population

Intervention developers and topic experts

Researchers / evaluators

Practitioners and policy-makers

Funders

**6. Implications (what else needs adapting?)**

For example, if an aspect of content is adapted, what does this mean for materials, training, technical assistance, delivery?

**7. Effect on theory of change (is the theory of change adapted by default?)**

Theory intact

Theory affected
